# Analysis of Bacterial and Fungal Infections after Cytoreduction Surgery and Hyperthermic Intraperitoneal Chemotherapy: An Observational Single-Centre Study

**DOI:** 10.1155/2019/6351874

**Published:** 2019-08-01

**Authors:** Talat A. M. Albukhari, Hanaa Nafady-Hego, Hamed Elgendy, Hanan M. Abd Elmoneim, Asmaa Nafady, Abdulaziz M. Alzahrani

**Affiliations:** ^1^Haematology and Immunology Department, Faculty of Medicine, Umm Alqura University, Mecca, Saudi Arabia; ^2^Microbiology and Immunology Department, Faculty of Medicine, Assiut University, Assiut, Egypt; ^3^Division of Translational Medicine, Sidra Medical and Research Center, Doha, Qatar; ^4^Anaesthesia Department, Faculty of Medicine, Assiut University, Assiut, Egypt; ^5^Department of Anaesthesia, King Abdullah Medical City, Mecca, Saudi Arabia; ^6^Department of Anaesthesia, Hamad Medical Corporation, Doha, Qatar; ^7^Weill Cornell Medicine, Doha, Qatar; ^8^Department of Pathology, Faculty of Medicine, Minia University, Minia, Egypt; ^9^Department of Pathology, Faculty of Medicine, Umm Alqura University, Mecca, Saudi Arabia; ^10^Department of Clinical Pathology, Faculty of Medicine, Assiut University, Assiut, Egypt; ^11^Department of Clinical and Chemical Pathology, Qena Faculty of Medicine, South Valley University, Qena, Egypt; ^12^Department of Surgery, King Abdullah Medical City, Mecca, Saudi Arabia

## Abstract

**Introduction:**

While hyperthermic intraperitoneal chemotherapy (HIPEC) after cytoreduction surgery (CRS) has been shown to improve patient survival and disease-free progression in peritoneal carcinoma (PC) patients, the procedure relates to a high postoperative infection rate. Herein, we report the bacterial and fungal infections after CRS and HIPEC from a single institution in Saudi Arabia.

**Patients and Methods:**

A prospective observational study was conducted on 38 patients with PC selected for CRS/HIPEC procedure between 2012 and 2015 in our centre.

**Results:**

Postoperative bacterial and fungal infection within 100 days was 42.2%, bacterial infection was reported always, and fungal infection was reported in 5 (13.2%) cases. Infections from the surgical site were considered the most common infection site. Multidrug-resistant extended-spectrum beta-lactamase (ESBL) *Escherichia coli* was the most frequent isolate, followed by multidrug-resistant *Acinetobacter baumannii* and *Pseudomonas aeruginosa*. Lower preoperative albumin and a prolonged preoperative activated partial thromboplastin time (APTT) are associated with postoperative infections, while a prolonged preoperative hospital stay (hazard ratio (HR) = 1.064; confidence interval (CI) = 1.002–1.112; *P*=0.042) and more intraoperative blood loss (>10%) (HR = 3.919; 95% CI = 1.024–14.995; *P*=0.046) were independent risk factors for postoperative infections. Three cases died during the follow-up period; all were due to infection.

**Discussion:**

The infection rate in our centre compared to previous studies of comparable patients was matching. Effective management of postoperative infections should be considered, and identified risk factors in this study can help to focus on effective prevention and treatment strategies.

## 1. Introduction

In the past, peritoneal carcinomas (PCs) were considered an untreatable situation, with poor prognosis and short life expectancy following diagnosis. Recently, cytoreduction surgery (CRS) with hyperthermic intraperitoneal chemotherapy (HIPEC) is being used more and more in selected PC patients and offers a promising prolonged survival or disease-free progression to PC patients [1, 2].

CRS combined with HIPEC was developed first by Spratt in 1980 as a locoregional treatment for primary or secondary peritoneal tumours. The surgical procedure for CRS/HIPEC is described elsewhere [3]. In summary, HIPEC after CRS rests on surgical removal of the primary tumour and applying a concentrated and heated chemotherapeutic agent in the peritoneal cavity. This procedure provides a high locoregional concentration of the chemotherapeutic agents with a low systemic effect. Critical care interventions are required for at least 48 hours to provide any postoperative organ or vasopressor support [4–6]. Mitomycin C (MMC) and oxaliplatin are both suitable as intraperitoneal chemotherapeutic agents in HIPEC for PC, and both showed encouraging survival results [7].

CRS/HIPEC has been widely existing for more than 20 years. However, this treatment has received a heavy criticism for its cost [8], high rates of potentially life-threatening complications as a result of the complexity of the combined procedure [9], and extraperitoneal spread of tumour [10], which accordingly resulted in limiting its general acceptance. Recently, after the increase of the experienced staff number globally, many institutes have reported encouraging results on patients' survival and disease-free interval after CRS combined with HIPEC in PC setting and hence may be considered as cost-effective management [8, 11–17]. Infections after CRS/HIPEC are the most common comorbidities that can lead to death [18, 19]. CRS/HIPEC technique is recently introduced in our area and led to treating many untreatable cases. The purpose of our work was to analyze the infections and determine their associated risk factors in patients with CRS and HIPEC procedures.

## 2. Patients, Materials, and Methods

### 2.1. Patients

All patients with PC who were eligible for CRS/HIPEC at King Abdullah Medical City, Mecca, Saudi Arabia, from March 2012 to May 2015, were included in our study, and the study was approved by the institutional review board (King Abdullah Medical City Ethics Committee).

### 2.2. CRS/HIPEC Procedure and Perioperative Management

The surgical procedure for HIPEC is described elsewhere [7]. In summary, HIPEC after CRS rests on surgical cytoreduction or macroscopic removal of the primary tumour followed by an administration of a highly concentrated MMC (12.5 mg/m^2^ dissolved in 1 litre normal saline) inside the peritoneal cavity. Heat is applied after an administration of the drug from 41 to 42°C for 90 min. Drains of a circuit pump placed inside the peritoneal cavity were used to extend and circulate the heated chemotherapy. HIPEC provides a high locoregional concentration of the chemotherapeutic agents with low systemic effect as the high temperature increases the infiltration ability of the drug without affecting the cell viability, and it also reduces side effects on the cardiovascular system, oxygen consumption, and coagulation. Herein, CRS/HIPEC surgeries were done by the same surgical team and using the same procedure. The hemodynamic status was controlled by using cooled infusions and replacement of fluid loss. To protect against postoperative infection, 2 gm cefazolin and 500 mg IV metronidazole were infused to patients, half an hour before surgery, and continued for 72 hours thereafter. Thoracic epidural analgesia was done to reduce the postoperative pain and the duration of postoperative ventilation. Immediate postoperative intensive care unit (ICU) admission for at least 48 hours was scheduled for all patients to provide postoperative organ or vasopressor support. Follow-up of patients was done monthly for the first 100 days and then every six months until the last visit or death. Our data included the first 100 days only. Clinical assessment and pathological investigation of tumour markers and CT scan's assessment were done to diagnose recurrence.

### 2.3. Identification of Bacterial and Fungal Infections in the Postoperative Period

Detection of pathogens was done as described before [20]. Samples of blood, stool, urine, sputum, pharyngeal swab, catheter tip, or any discharge were withdrawn by a puncture or through a proper drainage tube and were collected under complete aseptic conditions and examined twice a week. All samples were subjected to staining by Gram staining and cultured on conventional culture including mannitol salt agar, 5% chocolate agar, 5% sheep blood agar, and thioglycolate broth (Somatco, Riyadh, Saudi Arabia). Bacterial concentrations and identification were done by the Vitek system (bioMerieux Marcy l' Etoile, France) and by a single person; results were released after one week, and negative results are considered when there was a failure of bacterial growth after one week. For blood cultures, BacT/ALERT (bioMerieux) was used. Infections caused by either bacterial or fungal microorganisms were defined by the Centres for Disease Control and Prevention's National Nosocomial Infections Surveillance System [21, 22]. Bacteremia was divided into primary or secondary depending on detection of the definite source of infection. Primary bacteremia had no definite source of infection, while, secondary bacteremia, the same organism, was isolated from blood culture and from other definite site. In catheter-related bloodstream infection (CRBSI) setting, the catheter tip and the blood culture revealed the same isolated pathogen [23]. Two types of surgical site infection (SSI) are superficial and deep. Wound infections are superficial. Peritonitis and intra-abdominal abscess are deep. Fungal infection was defined by isolation of organisms on culture and temperature more than 38°C.

### 2.4. Management of Postoperative Infection

Empiric antimicrobial treatment in the form of cefazolin, cefuroxime, cefepime, imipenem, ciprofloxacin, erythromycin, tazocin, amikacin, and metronidazole was initiated until culture results were released. Antibiotics were later adjusted or changed by the culture results. In case of fungal infection, fluconazole was given at 5–10 mg/kg/day IV over 1 hour; if there was no improvement, amphotericin B was administered as a continuous drip at a rate of 0.25–1 mg/kg/day IV. Miconazole oral gel was used daily, and the wide-spectrum antibiotic was stopped.

### 2.5. Statistical Analysis

Quantitative data were analyzed by the Student's *t*-test, and qualitative data were analyzed by the chi-square test to differentiate between the two studied groups: group 1 (complicated with infections) and group 2 (not complicated with infections). Data are presented as mean ± standard deviation or percentage where appropriate. The potential of several preoperative, operative, and postoperative variables for postoperative infection was measured by Cox's proportional hazard regression models with a 95% confidence interval, and the variables that gave *P* value of less than 0.05 in univariate analyses were used in a logistic regression model to determine the independent risk. A statistically significant relationship was indicated by *P* value of less than 0.05 by using Package for the Social Sciences for Windows statistical software (version 21).

## 3. Results

### 3.1. Patient Characteristics

Herein, a total of 38 patients who underwent CRS/HIPEC procedures from March 2012 to May 2015 met the inclusion criteria and were included in our cohort prospective study. The mean age of patients was 52 ± 14 (24.5–74.9) years, and the median body mass index was 26.63 (16.41–40.42) kg/m^2^. The number of female patients was more than that of male patients: 23 and 15, respectively. Mucinous adenocarcinoma (15 (39.5%)) was the most commonly encountered histopathology; the colorectal tumour was the primary site of tumour in 20 (52.6%) patients. Tumour grading, organ metastasis, and KRAS mutation in the primary tumour were detected and compared between the studied groups. The total hospital stay follow-up period was (37.6 ± 38.6) (10–100) days, and the ICU stay was (4.9 ± 2.2; 2–11). Only new infections that occurred following the procedure were included; preoperative infections were excluded. There were only 3 mortalities, and all had an infection.  Table 1 shows the comparison between cases complicated with infections and those not complicated with infections.

### 3.2. Types of Infection and Pattern of Antibiotic Sensitivity of Pathogens

During the postoperative follow-up period, bacterial and/or fungal infection was reported in 16 (42.1%) cases, bacterial infection was reported always, and fungal infection was reported in 5 (13.2%) cases. Patients with infection showed 43 episodes of infection, and infection rate was 2.8 per patient. In total, 45 bacterial and fungal pathogens were isolated from 2 polymicrobial and 41 monomicrobial episodes. The bacterial isolates were 36 pathogens. Gram-negative isolates were higher than Gram-positive organisms (Gram-positive/Gram-negative ratio is 7/29). Extended-spectrum beta-lactamase (ESBL) *Escherichia coli* were the most frequent bacterial pathogens, followed by multidrug-resistant *Acinetobacter baumannii*, *Pseudomonas aeruginosa*, and *Enterococcus faecium* isolates. Vancomycin-resistant *Enterococci* were isolated from 2 samples. In total, 9 fungal infections were caused by 7 yeasts other than *Candida albicans* (YOTCA) species and 2 by *Candida albicans* isolates. The exact details of the pathogens, site, and time occurrence are in Tables 2–5. The details of antibiotic resistance rates of the isolated pathogens are listed in Tables 6 and 7.

### 3.3. Focuses of Infection

Patients' investigations revealed forty-three episodes of bacterial and fungal infections. Surgical site represented the major focus (SSI; *n* = 23, 53.5%), followed by respiratory tract (*n* = 10, 23.3%), urinary tract (*n* = 6, 14%), and bloodstream (*n* = 4, 9.3%).

### 3.4. Risk Factors Associated with Postoperative Infection

The univariate analysis showed that lower preoperative albumin, a prolonged preoperative activated partial thromboplastin time (APTT), a prolonged preoperative hospital stay, and more intraoperative blood losses were potential risk factors for the occurrences of postoperative infections. Those potential predictors that showed significance (*P* < 0.05) were further tested by multivariate analysis; a prolonged preoperative hospital stay (hazard ratio (HR) = 1.064; confidence interval (CI) = 1.002–1.112; *P*=0.042) and more intraoperative blood loss (HR = 3.919; 95% CI = 1.024–14.995; *P*=0.046) independently predicted occurrence of postoperative infection (Table 8).

## 4. Discussion

CRS and HIPEC are a promising therapeutic modality for PC patients; however, postoperative infections still represent the main cause for mortality, prolonged hospital stay, and healthcare costs in those patients [8, 9, 24]. As reported before, we found an association of infections and mortality as all died cases in the first 100 days had an infection [18, 19]. Herein, sixteen patients (42.2%) experienced at least one infectious complication which was considered comparable to reports from other centres with similar growing experience (12%–56%) [25–28] and higher than others as some studies showed lower infectious complications among CRS/HIPEC procedures ranging from 24% to 36% [18, 19]. The infection rate in our study was 2.8, all infected patients had one bacterial infection or more, and 13.2% of patients had fungal infections. The high infection rate may be attributed to the complexity of CRS/HIPEC procedure itself, the patients' poor medical condition, the abdominal cavity being a contaminated environment, and the impairment of the immune system either due to the tumour or to chemotherapeutic agents' toxicity [24, 25, 29]; which all together made patients with PC who underwent CRS/HIPEC procedures in our centre a potentially high-risk population for infections. A total of 44.6 % of isolated pathogens were multidrug-resistant pathogens: ESBL Escherichia coli, Acinetobacter baumannii, ESBL Klebsiella pneumoniae, and vancomycin-resistant enterococci. This finding explains the mortality reported here which raises the demand for prompt measures to prevent emerging resistant strains. YOTCA species represented 77.8% of fungal isolates. The relation of infection and mortality was demonstrated by Velasco and co-authors as out of 11 mortality cases, multidrug-resistant pathogens were isolated in three [30]; in the same study, similar to our finding, SSI was the most common focus of infection. All together, polymicrobial infection, higher incidence of multidrug-resistant strains, and YOTCA species can cause increased mortality. Therefore, we studied the potential risk factors for infection to identify patients at high risk for infection and provide special care for them. Prolonged preoperative hospital stay and more intraoperative blood loss (>10%) were independent factors for postoperative infections.Prolonged preoperative hospital stay increases risk of patients' exposure to hospital-acquired pathogens and alters their immune status as mentioned before. Other preoperative variables including lower preoperative albumin and a prolonged preoperative APTT were potential risk factors underlying infections. Previous admissions 6 months before the operation and chemotherapy history did not affect our results. Splenectomy was done in 2 patients, so it was difficult to assess its attribution to prognosis. Unlike previous reports, we could not find an association between surgical time and postoperative infection [19, 24]. Our cumulative data showed that although we do not have intraoperative 60 days mortality, we still have high mortality and infection rates, high incidence of multidrug-resistant bacteria, polymicrobial infections, and YOTCA infection in our patients. Infection and mortality rates can be minimized by increasing the number and experience of the surgical team; developing a selective team including infection control specialist, nutritionist, and ICU specialist is crucial to improving patient outcomes. Moreover, shortening the preoperative hospital stay and minimizing the intraoperative blood loss should be considered to improve the patients' outcome. The small number of patients, difficulty in identifying recurrence, and absence of major changes in the used prophylactic or empirical drugs are the limitations of this study. Our prospective work is to increase the number of patients and to study impacts of growing experience on postoperative infections.

## 5. Conclusion

Owing to the obtained results, we suggest the use of a standardized protocol for the infection prevention, monitoring, and treatment in all patients enrolled for cytoreductive surgery and HIPEC based on the use of antibiogram presented in Tables 6 and 7 for postoperative prophylaxis; using antifungal prophylactics for patients may help decrease fungal infection. Changing the type or the duration of prophylactic antimicrobial in accordance with the antibiotic sensitivity finding here is in demand.

## Figures and Tables

**Table 1:** Characteristics of 38 patients who underwent hyperthermic intraperitoneal chemotherapy (HIPEC) operation.

Variables	Complicated with infection	Not complicated with infection	*P* value
	*N* = 23	*N* = 15	
*Preoperative variables*			
Age (year)	50.3 ± 12	54.5 ± 16	0.184
Gender (male/female)	(8/7)	(7/16)	0.142
BMI	27.7 ± 5.7	25.8 ± 5.4	0.369
Preoperative hospital stay (days)	10.4 ± 11.7	2739 ± 10586	0.872
Hypertension	3	2	0.469
Diabetes mellitus	6	3	0.455
Renal disease	2	1	0.644
Liver disease	2	0	0.345
Lymph node involvement	10	3	0.05
Organ metastasis	15	7	0.13
KRAS mutation	6	0	0.33

*Preoperative laboratory variables*			
CK (U/L)	376.7 ± 440.4	440.9 ± 345	0.658
CK-MB (U/L)	21.7 ± 18.4	19.55 ± 114.9	0.289
Troponin (U/L)	0.037 ± 0.048	0.062 ± 0.14	0.71
White blood cells 10^9^/L	7.3 ± 2.2	6.7 ± 3.2	0.49
Red blood corpuscles ×10^12^/L	4.5 ± 1.3	4.6 ± 0.6	0.853
Haemoglobin (g/dL)	10.9 ± 1.4	11.7 ± 1.9	0.378
C-reactive protein (mg/L)	19.5 ± 2	7.9 ± 9.3	0.198

*Serum electrolytes level*			
Sodium (mmol/L)	135.2 ± 3.3	136 ± 2.6	0.567
Potassium (mmol/L)	3.9 ± 0.5	4.3 ± 0.3	0.263
Calcium (mg/dL)	8.6 ± 0.6	8.7 ± 0.5	0.161
Phosphorus (mg/dL)	3.5 ± 1	3.5 ± 1	0.237
Magnesium (mg/dL)	1.9 ± 0.3	1.6 ± 0.5	0.227
Chloride (mmol/L)	100.5 ± 4	102.3 ± 2.2	0.952
Gamma-glutamyltransferase (U/L)	72.5 ± 73	74.7 ± 122.9	0.614
Uric acid (mg/dL)	3.9 ± 1.8	4 ± 1	0.165

*Liver function markers*			
Alanine transaminase (IU/L)	37.1 ± 24.9	48.6 ± 39.5	0.578
Aspartate transaminase (IU/L)	28 ± 27.9	33.5 ± 34.9	0.789
Alkaline phosphatase (U/L)	146.7 ± 119.6	108 ± 53.7	0.785
Amylase (U/L)	61.9 ± 53.6	69.8 ± 30.3	0.065
Total bilirubin (mg/dL)	0.53 ± 0.49	0.34 ± 0.41	0.606
Conjugated bilirubin (mg/dL)	0.15 ± 0.23	0.1 ± 0.1	0.632
Albumin (gm/dL)	3 ± 0.5	3.3 ± 0.42	0.699
A/G ratio	1.2 ± 0.56	1.2 ± 0.54	0.531
Total protein (gm/dL)	6.1 ± 1.3	6.53 ± 1.5	0.465

*Tumour markers*			
Alpha feto protein (Ng/mL)	2.1 ± 1.3	2.9 ± 1.5	0.207
CA 125 U/mL	57.5 ± 92.2	40.1 ± 78	0.661
CA 15-3 U/mL	19.4 ± 9.5	16.4 ± 8.6	0.546
CA 19.9 U/mL	42.1 ± 30	20.7 ± 13.7	0.045
CEA ng/dL	32.7 ± 61.1	53.5 ± 152.2	0.64

*Coagulation factors*			
INR	1.1 ± 0.2	1.1 ± 0.2	0.441
Platelet count ×10^9^/L	327 ± 151	234 ± 144	0.433
Prothrombin time (sec.)	34.4 ± 8.7	34.4 ± 8.7	0.08
Fibrinogen (g/L)	2.6 ± 1.6	1.3 ± 0.14	0.208

*Renal function markers*			
Blood urea nitrogen	10.2 ± 4.7	12.2 ± 3.7	0.02
Creatinine (mg/dl)	0.7 ± 0.2	0.8 ± 0.23	0.15
Bicarbonate (mmol/L)	26 ± 3.2	26.3 ± 4.4	0.329
Lactate dehydrogenase (U/L)	207.7 ± 99.2	177.4 ± 83	0.05
Lipase (U/L)	162.7 ± 70.1	150.2 ± 57.6	0.658
Cholesterol (mg/dL)	72.9 ± 42.5	104.8 ± 56.6	0.813
LDL cholesterol (mg/dL)	38.7 ± 27.6	70.1 ± 46.5	
HDL cholesterol (mg/dL)	19.4 ± 11	21.5 ± 16.7	0.347
Triglycerides (mg/dL)	88.8 ± 55.4	86.4 ± 42.5	0.252
Random glucose (mg/dL)	150.7 ± 70.7	158.9 ± 82.7	0.755
Glycosylated haemoglobin	7.6 ± 2.2	8.2 ± 1.8	0.918

*Iron panel*			
Iron (*μ*mol/l)	20.5 ± 15.8	91.3 ± 92	0.047
Total iron-binding capacity (*μ*g/dL)	236 ± 150	346 ± 90	0.281
Ferritin (*μ*g/L)	192.8 ± 178.5	116.2 ± 136.5	0.615
Transferrin (*μ*g/L)	11.8 ± 8.9	30.5 ± 19	0.07

*Operative variables*			
Surgical time (hour)	651 ± 218	576 ± 173	0.296
Colloids transfusion (mL)	1528 ± 899	1250 ± 621	0.911
Packed red blood cells (unit)	3.3 ± 2.6	4.5 ± 4	0.187
Fresh frozen plasma (unit)	4.2 ± 3	4.3 ± 1.9	0.271
Fluid (25% albumin) transfusion (mL)	83.3 ± 28.9	50 ± 7	0.271
Plasma protein (mL)	906 ± 670	775 ± 448	0.288
Platelets (unit)	4.3 ± 1.6	4.8 ± 1.5	0.085
Blood loss (mL)	2177 ± 1313	1558 ± 1064	0.829

*Postoperative variables*			
Length of ICU stay days	12.8 ± 34.3	5.1 ± 2.4	0.924
Readmission number	3.5 ± 4	2.1 ± 2.8	0.988

BMI, body mass index; CK, creatine kinase; INR, international normalized ratio; LDL, low-density lipoprotein; HDL, high-density lipoprotein; ICU, intensive care unit.

**Table 2:** Onset and pathogens isolated from surgical site infections (SSIs) after hyperthermic intraperitoneal chemotherapy (HIPEC) operation.

	Superficial			Deep		
	Pathogen	Number	Time (day)	Pathogen	Number	Time (day)
Bacterial isolate		12 (27%)			9 (20%)	
Gram-positive organisms	*Enterococcus faecium* (VRE)	3 (1)	16 ± 9.5	*Enterococcus faecium* (VRE)	2 (1)	14.5 ± 17.68
Gram-negative organisms	*Pseudomonas aeruginosa*	1	34	*Escherichia coli* (ESBL)	5	10 ± 10.07
	*Escherichia coli* (ESBL)	1	2	*Escherichia coli*	2	17 ± 21.21
	*Escherichia coli*	1	5			
	*Proteus vulgaris*	2	24 ± 19.8			
	*Acinetobacter baumannii* (MDRO)	3	24 ± 12.29			
	*Klebsiella pneumoniae* (ESBL)	1	19			
Gram-positive/Gram-negative ratio		1/3				0.24
Fungal isolate		2 (4%)			2 (4%)	
	*Candida albicans*	1	25	*Candida albicans*	1	32
	YOTCA	1	58	YOTCA	1	39

VRE, vancomycin-resistant enterococci; ESBL, extended-spectrum beta-lactamase; MDRO, multidrug-resistant organisms; YOTCA, yeasts other than *Candida albicans*.

**Table 3:** Blood stream infections (BSI) after hyperthermic intraperitoneal chemotherapy (HIPEC) operation.

	Pathogen	Number	Time (day)
Bacterial isolate		2 (4%)	
Gram-negative organisms	*Acinetobacter baumannii* (MDR) (CRBSI, SSI)	1	18
	*Enterobacter cloacae* (CRBSI, SSI)	1	28
Gram-positive/Gram-negative ratio		0/2	
Fungal isolate		2 (4%)	
	YOTCA (UTI)	2	79 ± 5.46

MDRO, multidrug-resistant organisms; CRBSI, catheter-related bloodstream infection; SSI, surgical site Infection; YOTCA, yeasts other than *Candida albicans*; UTI, urinary tract infection.

**Table 4:** Respiratory tract infections (RTI) after hyperthermic intraperitoneal chemotherapy (HIPEC) operation.

	Pathogen	Number	Time (day)
Bacterial isolate		9 (20%)	
Gram-positive organisms	*Enterococcus faecium* (VRE)	1	91
Gram-negative organisms	*Stenotrophomonas maltophilia*	1	49
	*Pseudomonas aeruginosa*	5	42.4 ± 8.02
	*Acinetobacter baumannii* (MDRO)	1	18
	*Enterobacter cloacae*	1	4
		
		
Gram-positive/Gram-negative ratio		1/8	
Fungal isolate		1 (2%)	
	YOTCA	1	91

VRE, vancomycin-resistant enterococci; ESBL, extended-spectrum beta-lactamase; MDRO, multidrug-resistant organisms; YOTCA, yeasts other than *Candida albicans*.

**Table 5:** Urinary tract infections (UTI) after hyperthermic intraperitoneal chemotherapy (HIPEC) operation.

	Pathogen	Number	Time (day)
Bacterial isolate		4 (9%)	
Gram-positive organisms	*Enterococcus faecalis*	1	21
Gram-negative organisms	*Escherichia coli* (ESBL)	1	12
	*Acinetobacter baumannii*	1	52
	*Providencia stuartii*	1	60
Gram-positive/Gram-negative ratio		1/4	
Fungal isolate		2 (4%)	
	YOTCA	2	65 ± 21.2

ESBL, extended-spectrum beta-lactamase; YOTCA, yeasts other than *Candida albicans*.

**Table 6:** Antibiotic resistance rates of gram-positive isolates in patients after hyperthermic intraperitoneal chemotherapy (HIPC) operation.

Antibiotic	Number (%) of resistant isolates		
	*Enterococcus faecalis*	*Enterococcus faecium*	Total
	1	6	7
Ampicillin		4 (67%)	
Gentamicin			
Amikacin			
Ciprofloxacin			
Vancomycin		3 (50%)	
Penicillin G		3 (50%)	
Sulfamethoxazole/trimethoprim		1 (17%)	
Meropenem		1 (17%)	
Amoxicillin/clavulanic acid			
Clindamycin			
Erythromycin			
Oxacillin sodium			

**Table 7:** Antibiotic resistance rates of gram-negative isolates in patients after hyperthermic intraperitoneal chemotherapy (HIPC) operation.

Antibiotic	Number (%) of resistant isolates									
	ESBL *Klebsiella pneumoniae*	*Acinetobacter baumannii*	*Providencia stuartii*	*Pseudomonas aeruginosa* (CRP)	*Escherichia coli*	ESBL *Escherichia coli*	*Stenotrophomonas maltophilia*	*Enterobacter* species	*Proteus* species	Total
	1	6	1	6	3	7	1	2	2	29
Cefotaxime		4 (70%)						1 (50%)	1 (50%)	
Cefuroxime		3 (50%)			2 (50%)			1 (50%)	1 (50%)	
Ciprofloxacin	1 (100%)	5 (80%)		1 (11%)	3 (100%)	6 (85.7%)				
Gentamicin		5 (80%)		2 (33%)	3 (100%)	3 (42.9%)				
Nitrofurantoin			1 (100%)							
Piperacillin		1 (10%)		1 (11%)	3 (100%)				1 (50%)	
Piperacillin/tazobactam				1 (11%)	3 (100%)					
Sulfamethoxazole/trimethoprim	1 (100%)	4 (70%)			3 (100%)	5 (71.4%)				
Amikacin		5 (80%)								
Ceftazidime		5 (80%)		2 (33%)			1 (100%)	2 (100%)		
Cefepime		5 (80%)		1 (11%)	3 (100%)			1 (33%)	1 (50%)	
Imipenem		5 (80%)		4 (67%)						
Meropenem		4 (70%)		4 (67%)						
Amoxicillin/clavulanic acid			1 (100%)		2 (67%)			1 (50%)	1 (50%)	
Ampicillin			1 (100%)		2 (67%)			1 (50%)		
Levofloxacin				1 (11%)		3 (42.9%)	1 (100%)	2 (100%)		
Ceftriaxone		1 (10%)								
Colistin		1 (10%)								
Ceftriaxone		4 (70%)								
Cefotaxime				1 (11%)				1 (50%)		
Ampicillin/sulbactam										
Cefazolin										
Aztreonam										

**Table 8:** Univariable and multivariable analysis of infection in 38 patients after hyperthermic intraperitoneal chemotherapy (HIPEC) operation.

Variable	Univariable cox model			Multivariable cox model		
	HR	(95% CI)	*P* value	HR	(95% CI)	*P* value
Preoperative albumin/dL	0.323	(0.106–0.982)	0.046			
Preoperative activated partial thromboplastin time/sec	0.1.087	(0.998–1.183)	0.05			
Preoperative hospital stay/day	1.062	(1.014–1.112)	0.011	1.064	(1.002–1.112)	0.042
Interoperative blood loss >10%	3.696	(1.098–12.44)	0.035	3.919	(1.024–14.995)	0.046

HR, hazard ratio; CI, confidence interval.

## Data Availability

The data used to support the findings of this study are available from the corresponding author upon request.

## Abbreviations

APTT: Activated partial thromboplastin time
BMI: Body mass index
BSI: Bloodstream infection
CI: Confidence interval
CK: Creatine kinase
CRBSI: Catheter-related bloodstream infection
CRS: Cytoreduction surgery
ESBLs: Extended-spectrum beta-lactamases
HDL: High-density lipoprotein
HIPEC: Hyperthermic intraperitoneal chemotherapy
HR: Hazard ratio
ICU: Intensive care unit
INR: International normalized ratio
LDL: Low-density lipoprotein
MDRO: Multidrug-resistant organisms
MMC: Mitomycin C
PC: Peritoneal carcinoma
PTT: Partial thromboplastin time
RTI: Respiratory tract infection
SSI: Surgical site infection
UTI: Urinary tract infection
VRE: Vancomycin-resistant enterococci
YOTCA: Yeast other than *Candida albicans*.
